# Risk stratification utilizing sequential organ failure assessment (SOFA) score, antithrombin activity, and demographic data in sepsis-associated disseminated intravascular coagulation (DIC)

**DOI:** 10.1038/s41598-023-49855-y

**Published:** 2023-12-15

**Authors:** Toshiaki Iba, Cheryl L. Maier, Tomoki Tanigawa, Jerrold H. Levy

**Affiliations:** 1 Department of Emergency and Disaster Medicine, Juntendo University Graduate School of Medicine, 2-1-1 Hongo Bunkyo-Ku, Tokyo, 113-8421 Japan; 2 Department of Pathology and Laboratory Medicine, Emory University School of Medicine, Atlanta, GA USA; 3 Medical Affairs Section, Research & Development Division, Japan Blood Products Organization, Tokyo, Japan; 4 Department of Anesthesiology, Critical Care, and Surgery, Duke University School of Medicine, Durham, NC USA

**Keywords:** Biomarkers, Diseases

## Abstract

Disseminated intravascular coagulation (DIC) is a frequent complication in patients with sepsis and is associated with increased mortality. Anticoagulant therapy may be appropriate for certain patients with DIC, particularly those with increased disease severity and deficiency in the physiologic anticoagulant antithrombin. We retrospectively analyzed post-marketing survey data from 1562 patients with sepsis-associated DIC and antithrombin activity of 70% or less. All the patients were treated with antithrombin concentrates. Baseline sequential organ failure assessment (SOFA) score, DIC score, and antithrombin activity were assessed. Cox multivariate regression analysis, Kaplan–Meier curve analysis, and receiver operating characteristic (ROC) curve analysis were performed to evaluate the performance of variables used to assess mortality. Furthermore, a decision tree was constructed to classify the risk of 28-day mortality. COX multivariate regression analysis demonstrated a significant association of age, sex, baseline SOFA score, baseline antithrombin activity, and the presence of pneumonia or skin/soft tissue infection with increased mortality. The area under the curve of SOFA score or antithrombin activity for mortality was 0.700 and 0.614, respectively. Kaplan–Meier analysis demonstrated that mortality was significantly higher in patients with SOFA score ≥ 12 and antithrombin activity < 47%. The decision tree analysis accurately classified the risk of death into high (> 40%), medium (40%–20%), and low (< 20%) categories in 86.1% of the cohort. Twenty eight-day mortality can be strongly predicted using baseline SOFA score, antithrombin activity, infection site, age, and sex as variables in the clinical decision tree for patients with sepsis-associated disseminated intravascular coagulation (DIC).

## Introduction

Disseminated intravascular coagulation (DIC) is a frequent and critical complication of sepsis, and the mortality of sepsis patients increases considerably when DIC develops^1^. A multicenter cohort study reported that the prevalence of DIC in septic ICU patients was 50.9%, and the mortality rate of those patients with DIC was significantly higher than those without DIC (24.8% vs. 17.5%)^2^. Multiple scoring systems are available to aid in diagnosing and monitoring critically ill patients with DIC^3–5^. However, since the performance of scoring systems for accurately predicting outcomes has not been high enough, more simple and pragmatic methods for risk assessment are warranted. Recent literature highlights that the appropriate diagnosis of DIC is important for evaluating the disease severity and selecting the optimal anticoagulant therapy^6,7^. Sequential Organ Failure Assessment (SOFA) score is routinely used for monitoring the severity of sepsis in the ICU, and its superior performance over DIC scores in terms of outcome prediction has been reported^8^. In sepsis-associated DIC, the usefulness of coagulation markers such as prothrombin time and fibrinogen have been established, and these tests are included in the diagnostic criteria^9^. Additional biomarkers are of interest and likely clinically useful, particularly those reflecting endothelial damage, given the known derangement of endothelial function as an essential part of DIC^1^. Thus, the non-overt DIC scoring system released from the International Society on Thrombosis and Haemostasis (ISTH) included antithrombin activity, although the value of adding antithrombin remains uncertain^1^. Nonetheless, our intention is not to introduce a new biomarker to the DIC diagnostic criteria but to enhance prognostication in DIC by employing antithrombin activity for stratification.

Antithrombin is the most abundant and essential natural anticoagulant, inhibiting multiple coagulation factors, particularly thrombin and factor Xa^10^. A number of studies have revealed an association between reduced antithrombin levels and an increased risk of morbidity and mortality^8,11,12^. In addition, antithrombin activity has served as a marker of disease severity in DIC^13^. Notably, antithrombin supplementation has been reported to be effective only in patients with decreased antithrombin activity, and Hayakawa et al.^14^ indicated that the threshold was 43% or less. The primary mechanism for decreased antithrombin is considered to be related to increased vascular permeability, and the ISTH/DIC Scientific and Standardization Committee has proposed antithrombin activity as a marker for endotheliopathy^15^. In this study, we hypothesized that the inclusion of antithrombin activity and other factors in a stepwise classification may be useful. The objective of this study is to develop a practical procedure for identifying the suitable target for antithrombin supplementation by categorizing patients with an estimated mortality rate ranging from 20 to 40%.

## Materials and methods

### Patient cohort and data collection

Data was collected through post-marketing surveillance conducted by the Japan Blood Products Organization at 213 hospitals in Japan. The survey underwent review by the Institutional Review Board of each participating hospital and was conducted in accordance with the principles outlined in the Declaration of Helsinki and Good Post-Marketing Study Practice. This study was approved by the Research Ethical Review Committee of the Japan Blood Products Organization, and the study received Ethical approval from Juntendo University. Due to the anonymous nature of the data, the need for informed consent for the publication was waived by the Institutional Review Board of Juntendo University.

**Figure 1:**
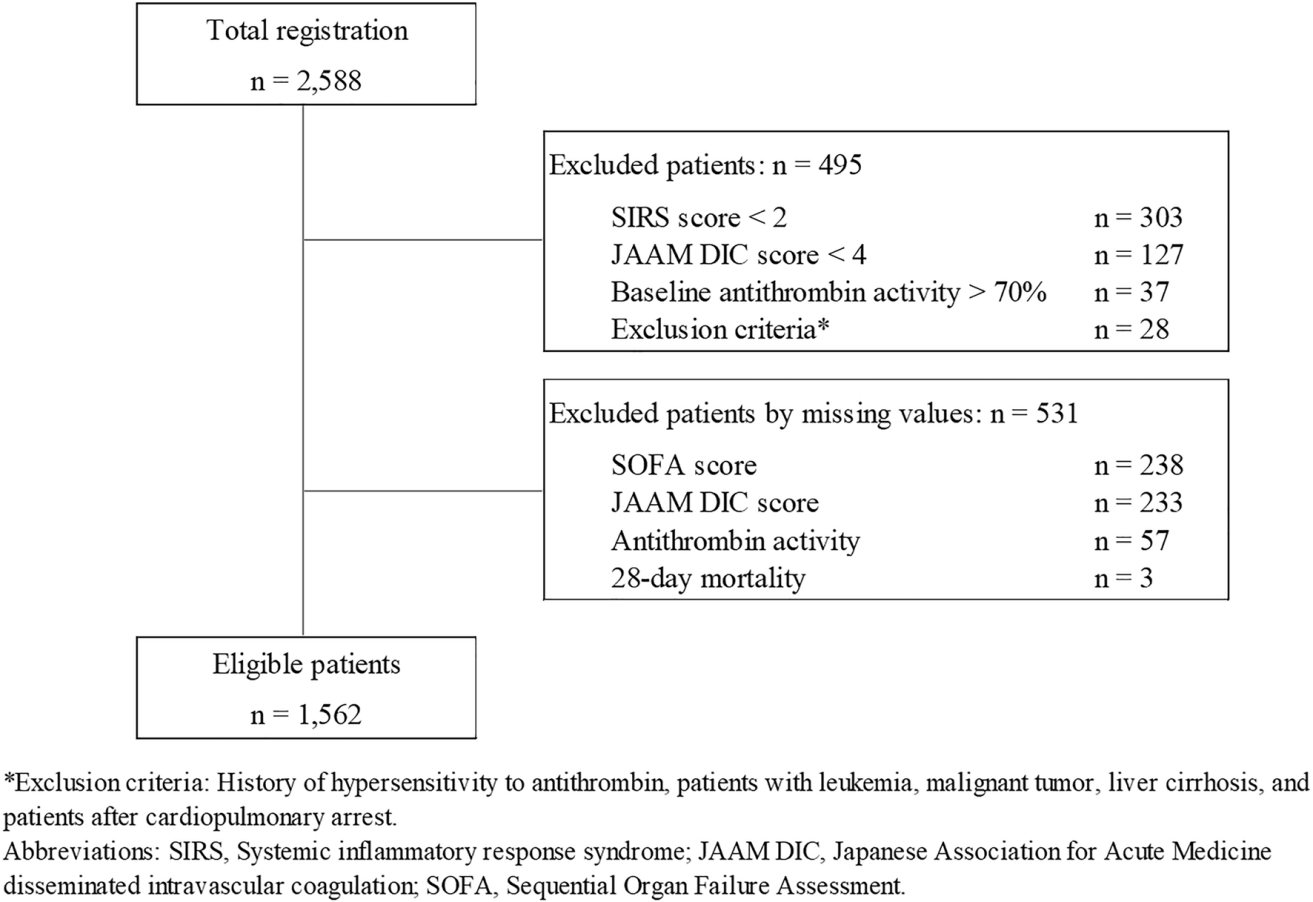
Patient selection. The data from the patients with sepsis-associated DIC with antithrombin activity of 70% or less and treated with antithrombin concentrate were utilized. Regarding antithrombin activity, sequential organ failure assessment (SOFA) score, and Japanese Association for Acute Medicine (JAAM) disseminated intravascuclar coagulation (DIC) score, when the data are absent, these data are categorized as 'missing data'.

Patients with sepsis-associated DIC based on the Japanese Association for Acute Medicine (JAAM) DIC criteria and treated with antithrombin concentrate (Neuart®; Japan Blood Products Organization, Tokyo) between April 2013 and April 2016 were analyzed retrospectively (n = 1562). Patients with antithrombin concentrate allergy, leukemia, malignancy, cirrhosis, or after cardiopulmonary arrest were excluded. In addition, missing values for SOFA score, JAAM DIC score, antithrombin activity, and 28-day mortality were also excluded from the study. Inclusion criteria included a JAAM DIC score of 4 or more^4^ and antithrombin activity of 70% or less (Fig. 1).

Baseline data, prior to antithrombin concentrate supplementation, was collected and included DIC score, SOFA score, and antithrombin activity. When multiple assessments were conducted, the worst score for each day was recorded. In the majority of cases, a daily administration of 1500 IU of antithrombin was given for three consecutive days in accordance with the regulation of the Japanese healthcare system. However, the duration of treatment could be extended at the discretion of each physician. Based on clinical judgment, the dosage of antithrombin was also permitted to be adjusted, either increased or decreased. There were no restrictions on the concomitant use of heparin, protease inhibitors, recombinant thrombomodulin, or other anticoagulants.

### Statistical analysis

Baseline patient demographics were compared between survivors and non-survivors. Continuous variables were presented as means with standard deviations, and T-tests were used to determine statistical significance. Categorical variables were presented as frequency distributions and percentages, and chi-square tests were used to determine significance.

Univariate Cox regression was performed to examine the risk of death up to day 28, and the references were used as the first category for categorical variables. To account for potential correlations and collinearity between variables, an initial multivariate Cox regression analysis was performed to narrow down the selection of variables. The variable selection method was stepwise, with α = 0.25 as the inclusion criterion and α = 0.15 as the exclusion criterion.

Kaplan–Meier curves were constructed to evaluate cumulative mortality from day 0 to day 28. Cox regressions were conducted to compare Kaplan–Meier curves between the two groups. Receiver operating characteristic (ROC) curve analysis was performed to assess the ability of baseline antithrombin activity and SOFA score to predict 28-day survival, and the area under the curve (AUC) was calculated.

Recursive partitioning with binary cuts of the entered variables was employed to develop the decision tree. The best differentiator between treatment failure and success was selected as the root of the tree. This process was repeated iteratively until the optimal discrimination between success and failure in classification was achieved.

After the fixation of the decision tree model, the performance was validated in the different cohorts constructed in the other post-marketing survey^9,11^. Data for validation was constructed from multi-institutional surveys performed between 2014 and 2016. A total of 926 patients with sepsis-associated DIC, antithrombin activity with less than 70%, and treated with antithrombin concentrate of 1500 IU/day for three consecutive days were registered in the survey.

A predictive model was constructed using logistic regression analysis, and its performance was evaluated using ROC analysis.

Results are reported as Hazard ratio (HR), 95% confidence interval (CI), and P values. Statistical analyses were performed using SAS (version 9.4, SAS Institute, Co., Ltd., Cary, NC, USA) for Cox regression analysis and R (version 4.1.1, R Foundation for Statistical Computing, Vienna, Austria) for the others.

### Ethical approval

The survey underwent review by each of the 213 Institutional Review Boards of participating hospitals and was conducted with the ethical standards laid down in the 1964 Declaration of Helsinki and its later amendments.

### Consent to participate

The survey was conducted as a post-marketing survey, and the treatment was administered in accordance with the recommendations of the Japanese Clinical Practice Guidelines for the Management of Sepsis and Septic Shock. Due to the anonymous nature of the data, the need for informed consent was waived by the Institutional Review Board of Juntendo University.

## Results

Table 1 summarizes the patient demographic data. In this study, 2,588 patients were initially registered. However, 495 cases did not fulfill the criteria or met the exclusion criteria, and data from an additional 531 cases were excluded due to the missing values. Among the 1562 patients, 1298 survived (83.1%), while 264 (16.9%) died. The proportion of females was significantly higher among the survivors (*P* = 0.001), and the mean age of the survivors was significantly lower than that of the non-survivors (*P* < 0.001). The mean SOFA score was significantly lower in survivors (8.9 vs. 11.7, *P* < 0.001), while the mean JAAM/DIC score was not different between survivors and non-survivors. The mean baseline antithrombin activity was higher in survivors (48.5% vs. 43.5%, *P* < 0.001). Pneumonia was more frequent in non-survivors (*P* < 0.001), while pyelonephritis and biliary tract infection were less common. The baseline SOFA scores of the cardiovascular system in survivors and non-survivors were 2.07 and 2.66, and those of the respiratory system were 1.70 and 2.27. Approximately half of the patients required mechanical ventilation, one-third of patients required renal replacement therapy, and more than 60% received catecholamine support. In this study, bleeding was observed in 77 (4.93%) of 1562 patients. Of these, 72 (4.61%) were major bleedings, and 12 (0.77%) were considered possibly related to antithrombin supplementation.

**Table 1:** Baseline characteristics of the patients.

Factor	Survivor		Non-survivor		*P* value
	1298		264		
Sex = male (%)	744	(57.30)	180	(68.20)	0.001
Age [mean (SD)]	69.78	(15.50)	73.46	(13.00)	< 0.001
Body weight [mean (SD)]	55.72	(13.19)	56.27	(15.03)	0.549
SOFA score [mean (SD)]	8.86	(3.73)	11.72	(3.94)	< 0.001
JAAM DIC score [mean (SD)]	5.62	(1.36)	5.63	(1.28)	0.918
Antithrombin activity [mean (SD)]	48.49	(12.12)	43.45	(13.01)	< 0.001
Source of infection					
Pneumonia (%)	242	(18.6)	101	(38.3)	< 0.001
Digestive tract (%)	333	(25.7)	59	(22.3)	0.293
Pyelonephritis (%)	193	(14.9)	19	(7.2)	0.001
Biliary tract (%)	111	(8.6)	12	(4.5)	0.038
Skin/soft tissue (%)	87	(6.7)	27	(10.2)	0.060
Urinary tract (%)	29	(2.2)	3	(1.1)	0.363
Intravenous catheter (%)	47	(3.6)	6	(2.3)	0.359
Others (%)	77	(5.9)	13	(4.9)	0.620
Unknown (%)	169	(13.0)	66	(25.0)	< 0.001
Antithrombin concentrate					
Daily dose (IU) [mean (SD)]	1571.4	(452.90)	1594.50	(434.80)	0.447
Duration (day) [mean (SD)]	3.18	(1.92)	3.46	(1.82)	0.027
Total dose (IU) [mean (SD)]	4709.5	(1906.70)	5304.1	(2307.58)	< 0.001
Co-administration					
Heparin (%)	195	(15.0)	47	(17.8)	0.296
Protease inhibitor (%)	267	(20.6)	53	(20.1)	0.922
Thrombomodulin (%)	752	(57.9)	162	(61.4)	0.336
Renal replacement therapy (%)	342	(26.3)	125	(47.3)	< 0.001
Mechanical ventilation (%)	643	(49.5)	193	(73.1)	< 0.001

*SD* Standard deviation, *SOFA* Sequential Organ Failure Assessment, *JAAM DIC* Japanese Association for Acute Medicine disseminated intravascular coagulation.

**Figure 2:**
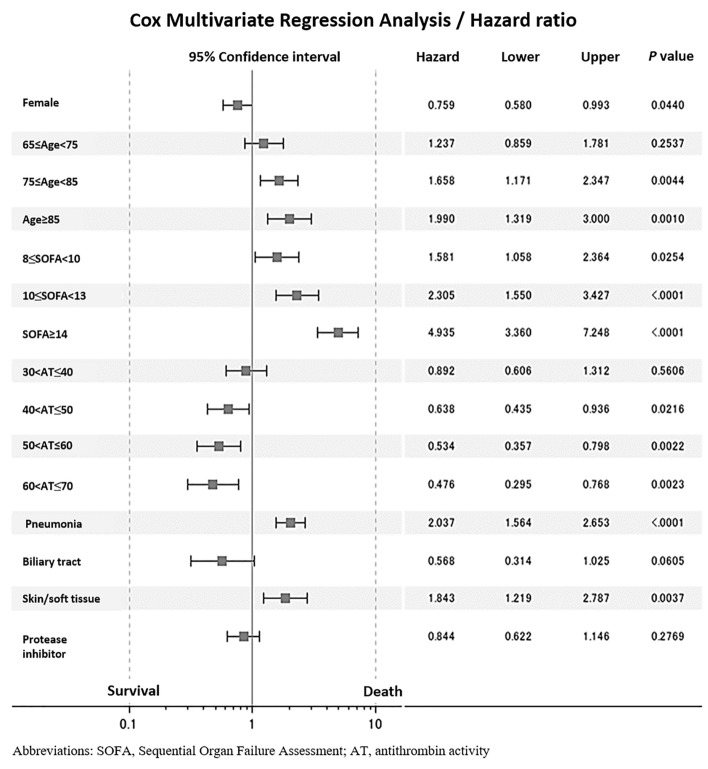
Cox multivariate regression analysis for survival factors. Cox multivariate regression analysis identified male sex, age 75 years or older, baseline sequential organ failure score (SOFA) score ≥ 8, and the presence of pneumonia or skin/soft tissue infection as significant factors associated with worsened survival. On the other hand, baseline antithrombin activity > 40% was found to be associated with better survival.

Cox multivariate regression analysis following the univariate regression analysis (Suppl. Fig. 1) demonstrated that sex, age, baseline SOFA score, baseline antithrombin activity, and the presence of pneumonia or skin/soft tissue infection were significant factors that influenced survival (Fig. 2).

**Figure 3:**
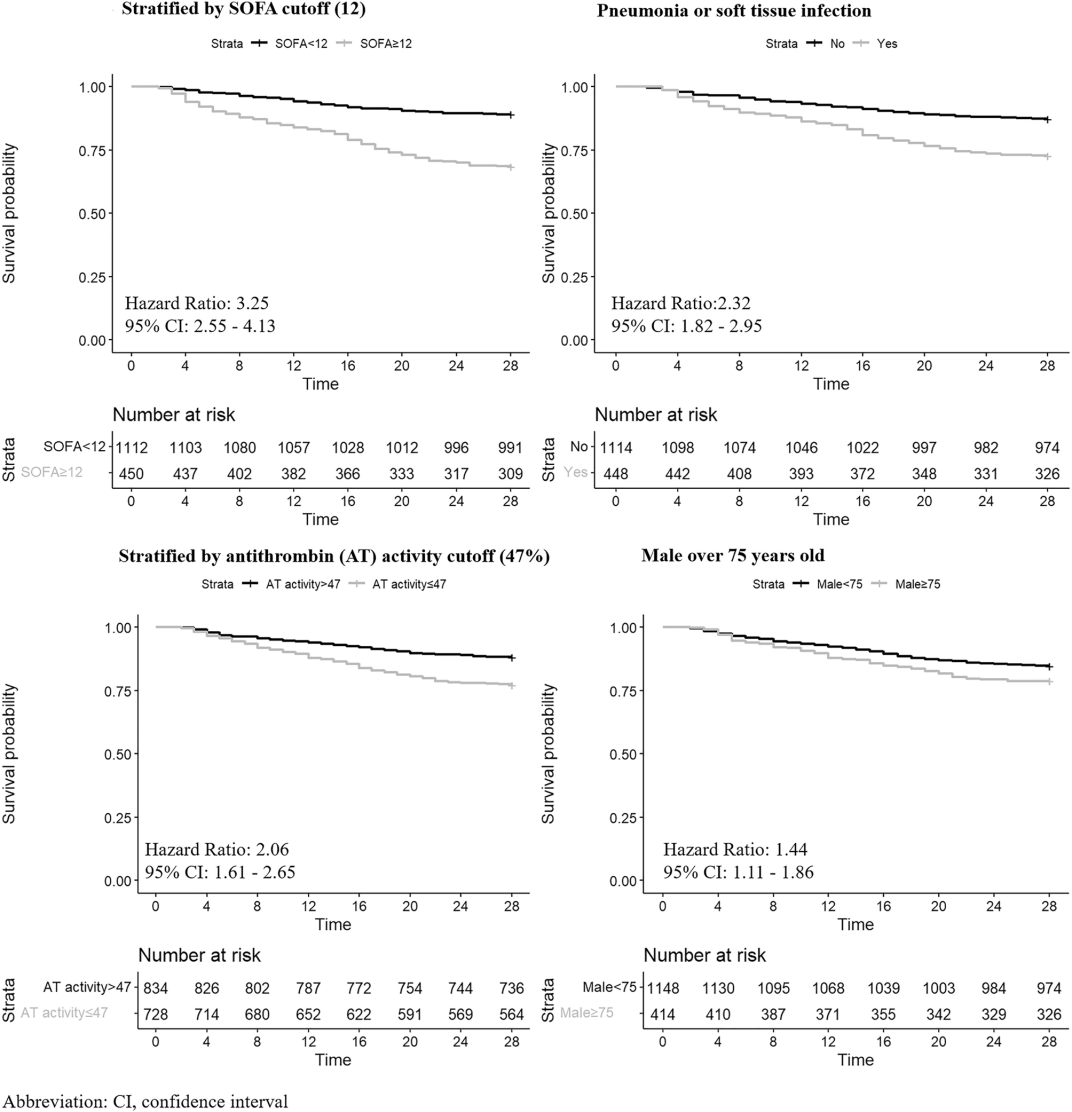
Kaplan–Meier curves for the cumulative survival of the patients with sepsis-associated DIC. Patients were divided into high (≥ 12) and low (< 12) sequential organ failure assessment (SOFA) score groups, high (> 47%) and low (≤ 47%) antithrombin activity groups, presence or absence of pneumonia or soft tissue infection, and male sex and older than 75 years old or no. Significant differences were seen between the groups.

Kaplan–Meier curves demonstrated a difference in 28-day survival between patients with a baseline SOFA of 12 or more versus those with a baseline SOFA of less than 12 and between patients with baseline antithrombin activity of 47% or less and those with a baseline antithrombin activity or more than 47%. Patients with pneumonia or skin/soft tissue infection and males 75 years old and over showed worse outcomes (Fig. 3).

**Figure 4:**
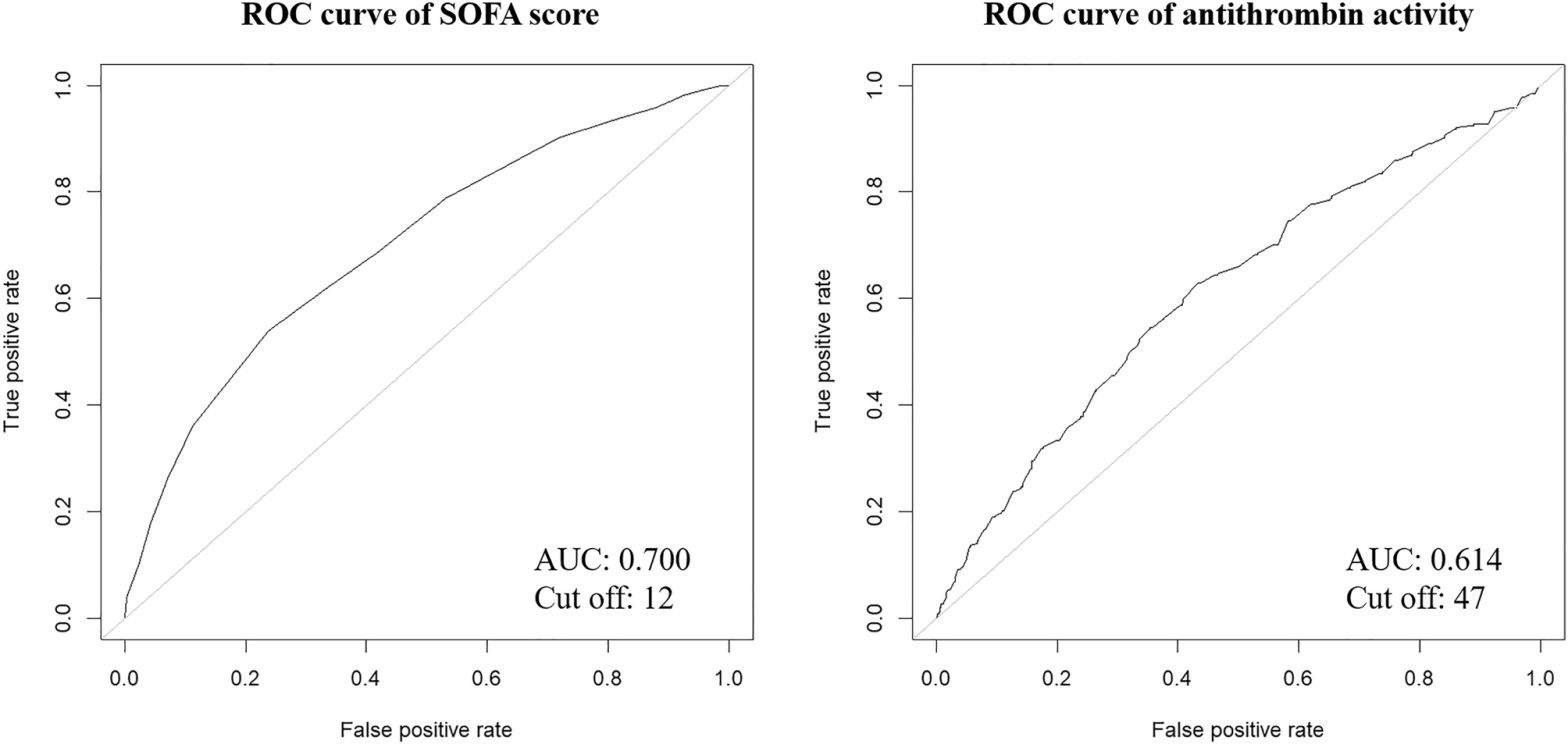
Receiver operating characteristic curves of the SOFA score and antithrombin activities for 28-day survival. Receiver operating characteristic (ROC) curves of the baseline sequential organ failure assessment (SOFA) score and baseline antithrombin activity for 28-day survival are depicted. The respective areas under the ROC curves (AUCs) were 0.700 for the SOFA score and 0.614 for the antithrombin activity.

Figure 4 shows ROC curves of baseline SOFA score and antithrombin activity for survival. The AUCs of the SOFA score and antithrombin activities were 0.700 and 0.614, respectively. Cutoff values of the SOFA score and antithrombin activity were 12 and 47%, respectively.

**Figure 5:**
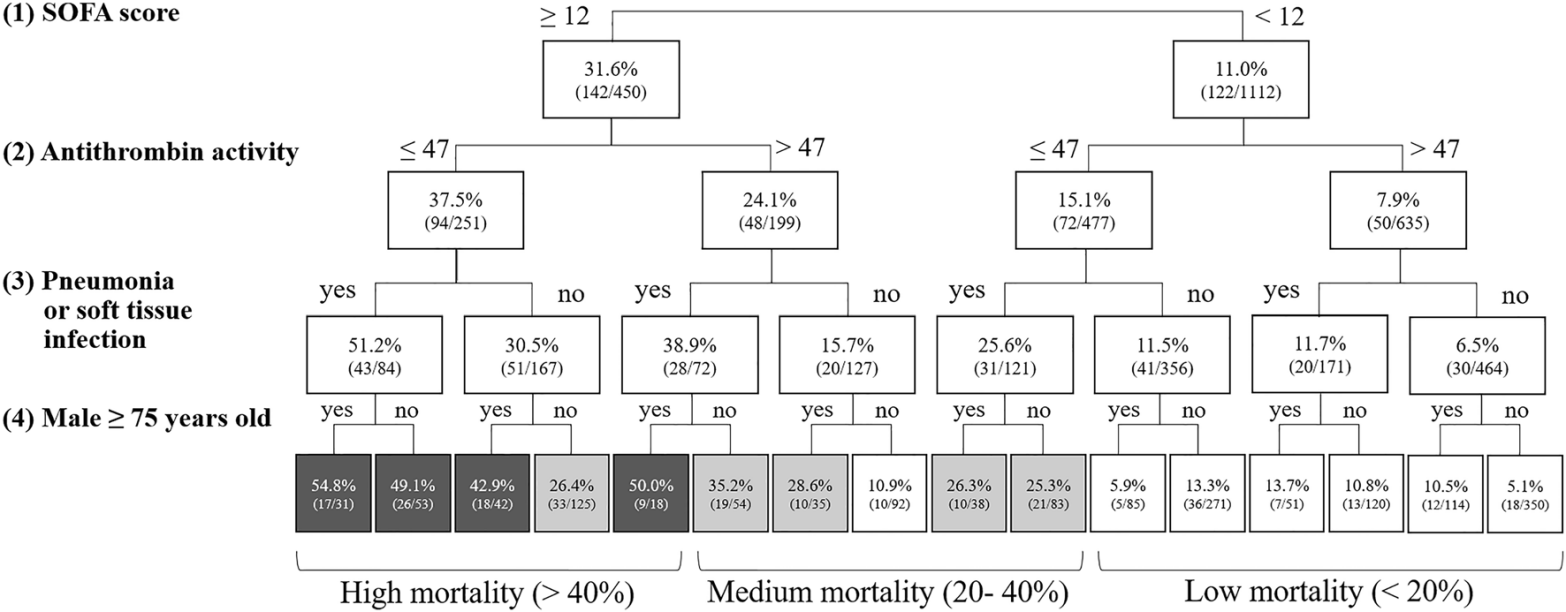
Decision tree model for risk stratification. A decision tree model was used to categorize patients to either 28-day mortality of high (> 40%), medium (20–40%), and low (< 20%) range according to the sequential organ failure assessment (SOFA) score, baseline antithrombin activity, infectious site, sex, and age. Two categories (13.9% of the total cases) were not appropriately classified.

A decision tree model analysis constructed using SOFA score, baseline antithrombin activity, infectious site, sex, and age classified the patients for 28-day mortality as high (> 40%), medium (20–40%), or low (< 20%) range. Two categories representing a minority of the total cases (13.9%) were not appropriately classified. These included: (1) SOFA score of 12 and higher, antithrombin activity greater than 47%, not having pneumonia or skin/soft tissue infection, and being female or male under the age of 75; and (2) SOFA score of 12 and higher, antithrombin activity 47% and less, not having pneumonia or skin/soft tissue infection, and being female or male under the age of 75 (Fig. 5). After excluding the missing data, 732 data sets were available in the validation cohort (28-day survival, 73.8%). Among 732 cases used for validation, 616 (84.2%) matched the estimated mortality, while 116 (15.8%) did not.

**Figure 6:**
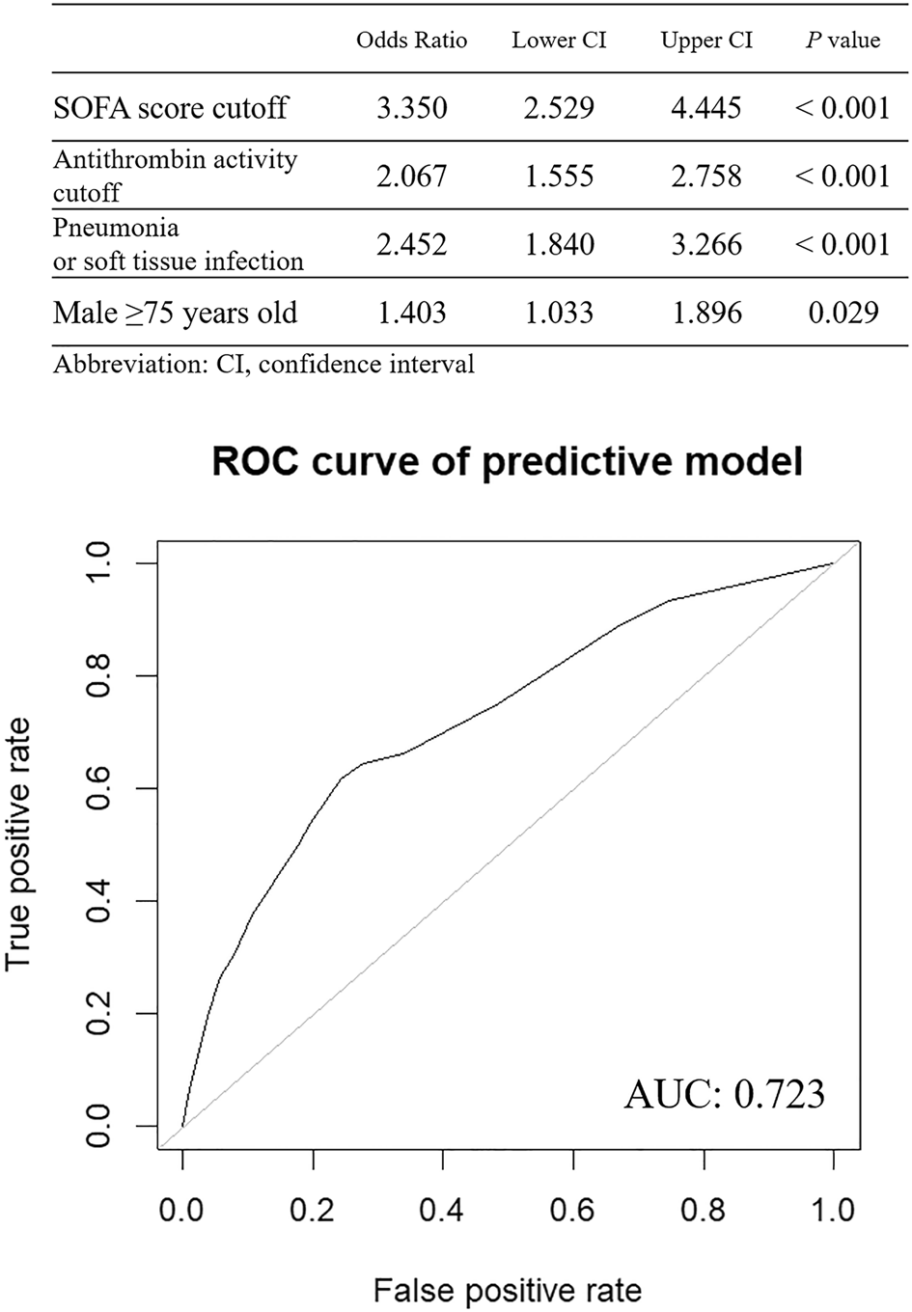
Evaluation of a predictive model using decision tree risk factors. A predictive model was constructed using logistic regression, and its performance was evaluated using Receiver operating characteristic (ROC) analysis. Four decision tree variables were used in the prediction model, and the objective variable was 28-day mortality. The predictive score was constructed as 3.350 * SOFA cutoff + 2.067 * antithrombin activity (AT) cutoff + 2.452 * infection site + 1.403 * male and ≥ 75 years old. The results of ROC analysis showed high predictive ability with an area under the curve (AUC) of 0.723.

High mortality was associated with SOFA score of 12 or more, baseline antithrombin activity of 47% or less, presence of pneumonia or skin/soft tissue infection, and male patients aged 75 or more. The predictive score constructed using these factors demonstrated predictive ability with an AUC of 0.723 (Fig. 6). Hence, the information related to infectious sites, sex, and age does not appear to exert a significant influence on the accuracy of mortality prediction.

## Discussion

Patients with sepsis may experience coagulation activation initiated by the expression of tissue factor and phosphatidylserine on monocytes/macrophages and accelerated fibrin formation on the surface of activated platelets^16,17^. At the same time, monocytes/macrophages promote intravascular inflammation, leading to systemic vascular endothelial damage and disseminated microthrombus formation^18^. Endothelial cells are critical regulators of vascular permeability, and endothelial damage with loss of appropriate barrier function is an essential part of the pathophysiology of DIC^3^.

In the present study, Cox regression analysis revealed a significant association between patient age, sex, underlying disease, SOFA score, and baseline antithrombin activity with overall outcome. A threshold value of 47% for baseline antithrombin activity was established for predicting an increased risk of death, which is consistent with previous reports^11,14^. However, the area under the ROC curve (AUC) showed discriminative power for baseline antithrombin activity of 0.614, which may not be sufficiently high. It is important to note that although antithrombin activity has predictive value for patient outcomes in sepsis-associated DIC, it is just a part of the complex pathophysiological mechanisms involved. Therefore, it is crucial to combine it with other relevant parameters for a more precise assessment. In addition to antithrombin activity, our study also included SOFA score, patient characteristics, and information about underlying diseases for the prognostic assessment. Although stratification by adding infectious sites age, and sex did not show a strong impact on the improvement of mortality prediction, the performance of categorizing the population of 20–40% mortality improved from 71.2 to 86.1%.

Antithrombin supplementation via concentrates is recommended in the Japanese guidelines for sepsis management and is commonly performed in Japan^19^. A major goal of this current study was to find an appropriate target value for initiating antithrombin therapy. Using acquired antithrombin-deficient plasma, Tsuchida et al.^20^ demonstrated thrombin generation capability depends on the antithrombin activity. The study demonstrated that the amount of thrombin generation exhibited significant changes when antithrombin activity reached 50% or lower, with particularly notable generation observed when antithrombin activity dropped below 30%. Notably, the prognosis was poor in patients with extremely low antithrombin activity due to organ failure associated with widespread microvascular thrombosis.

Other than low antithrombin activity, a Japanese nationwide observational study demonstrated that patients with sepsis-associated DIC and high disease severity are the optimal targets for anticoagulant therapy^21^. Another multicenter observational study demonstrated that survival benefits associated with anticoagulant therapies were also found in DIC patients with high severity of illness (SOFA score, 13 to 17; adjusted HR 0.601; 95% CI 0.451 to 0.800)^22^. Since the effect of anticoagulant therapy is presumed to be more significant in severe cases, our intention was to stratify the patients based on mortality. The results of the present study demonstrate that stepwise classification using SOFA score, antithrombin activity, underlying disease, sex, and age enables the identification of patients with relevant severity possible. Selecting the appropriate target population is vital for planning clinical studies, and patients with too severe or not severe enough disease are not appropriate for inclusion. For example, for selecting 28-day mortality of 20% to 40% (medium severity), if the SOFA score is less than 12, the antithrombin activity should be 47% or less, and the underlying diseases should include pneumonia or skin/soft tissue infection. If the SOFA score is 12 or more, antithrombin should be greater than 47%, and male patients aged 75 or over should be excluded. It is possible to make the patient selection by a single indicator; for example, selecting patients with SOFA scores of a particular range (one step) or a combination of SOFA score and antithrombin activity (two-step). If using time-sequential data, the selection may be more precise (Suppl. Figs. 2 and 3). However, considering that the present four-step system includes information on patient demographic data and underlying disease, the proposed system can select specific targets more precisely and is, therefore, aligned with personalized medicine principles.

Although the decision tree is a more simplified model than what is currently used, it allows for the simulation of complex processes to facilitate decision-making^23^. The decision tree analysis is not considered highly robust due to its relatively simplistic predictive algorithm, which lacks solid statistical logic. Thus, validation in another clinical cohort is important, and in this study, we examined the performance of the present decision tree in different septic patients with DIC. The validation cohorts were diagnosed as DIC and showed baseline antithrombin activity is less than 70% and were treated with antithrombin^9,11^. Consequently, a nearly equal percentage of patients matched the estimated mortality rate (derivation cohort: 86.1% vs. validation cohort: 84.2%). Therefore, the estimation performance was almost the same as that of the present study.

There are some limitations in our study. First, all patients had antithrombin activity of 70% or less, and patients with normal antithrombin activity were not included. The mean and standard deviation of antithrombin activity of the survivors at baseline were 48.5% and 12.1%, respectively, with an expectation that not many patients with DIC showed antithrombin activity of more than 70%. Matsubara et al.^8^ examined the relationship between antithrombin activity and mortality in sepsis patients and showed that mortality increased as the antithrombin activity decreased only when the activity was below 70%. In contrast, the mortality was low and consistent when the activity was above 70%. Nevertheless, a study that includes patients with an antithrombin activity of more than 70% is necessary. Second, the supplemented dose of antithrombin was 1500 IU/day for 3 days, which is lower than that used in other countries. This dosage regimen adheres to the regulations of the Japanese healthcare system but lacks support from clinical evidence. Third, a potential risk of bias exists as a substantial proportion of patients were excluded due to missing values for either the SOFA score or DIC score. Finally, all patients included in this study were treated with antithrombin concentrates, and additional studies inclusive of patients without antithrombin supplementation are required in order to generalize the results to all critically ill patients with sepsis-associated DIC.

## Conclusions

Risk stratification is crucial for estimating mortality and discerning the appropriateness of anticoagulant therapy. A stepwise classification using a decision tree with variables such as baseline SOFA score, antithrombin activity, underlying disease, sex, and age, provides a simple and straightforward modality for outcome stratification. This type of approach is expected to be widely utilized in both clinical practice and research settings, with the ultimate goal of not only predicting mortality but also guiding interventions to mitigate poor outcomes.

### Supplementary Information

Supplementary Figure 1.

Supplementary Figure 2.

Supplementary Figure 3.

Supplementary Information 1.

## Data Availability

The datasets generated during and/or analyzed in the current study are not publicly available due to contractual agreements with the participating institutes.
