# Supplementary material for: Risk stratification utilizing sequential organ failure assessment (SOFA) score, antithrombin activity, and demographic data in sepsis-associated disseminated intravascular coagulation (DIC)
Source: Sci Rep. 2023 Dec 15;13:22502. doi: 10.1038/s41598-023-49855-y (PMC10728127; doi:10.1038/s41598-023-49855-y)
Supplement: Supplementary file 2 — Supplementary Figure 2. [file 41598_2023_49855_MOESM2_ESM.docx]

**Supplementary figure 2. The changes in the SOFA score**

Changes in the mean SOFA score ± standard deviation in the survivor and non-survivor groups are plotted. The survivor showed lower SOFA scores at baseline, Day 3, and 6.


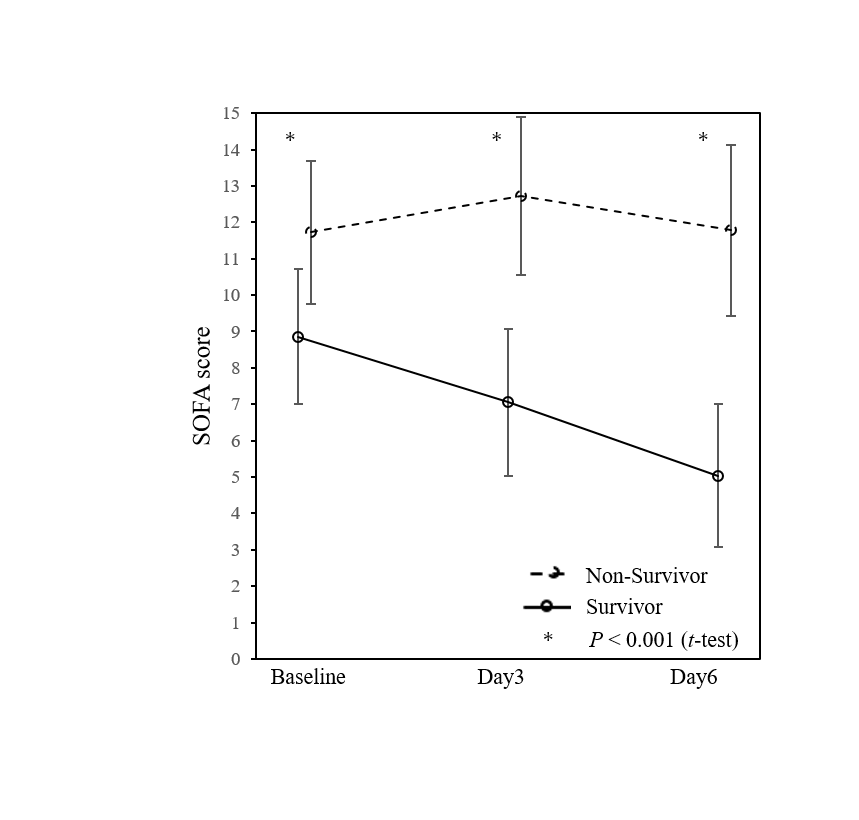


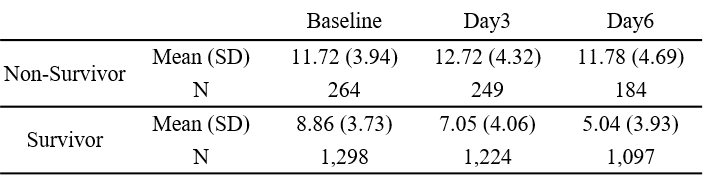


Abbreviation: SD, Standard Deviation.
