# Supplementary figures and images for: Risk stratification utilizing sequential organ failure assessment (SOFA) score, antithrombin activity, and demographic data in sepsis-associated disseminated intravascular coagulation (DIC)
Source: Sci Rep. 2023 Dec 15;13:22502. doi: 10.1038/s41598-023-49855-y (PMC10728127; doi:10.1038/s41598-023-49855-y)

## Slide 1
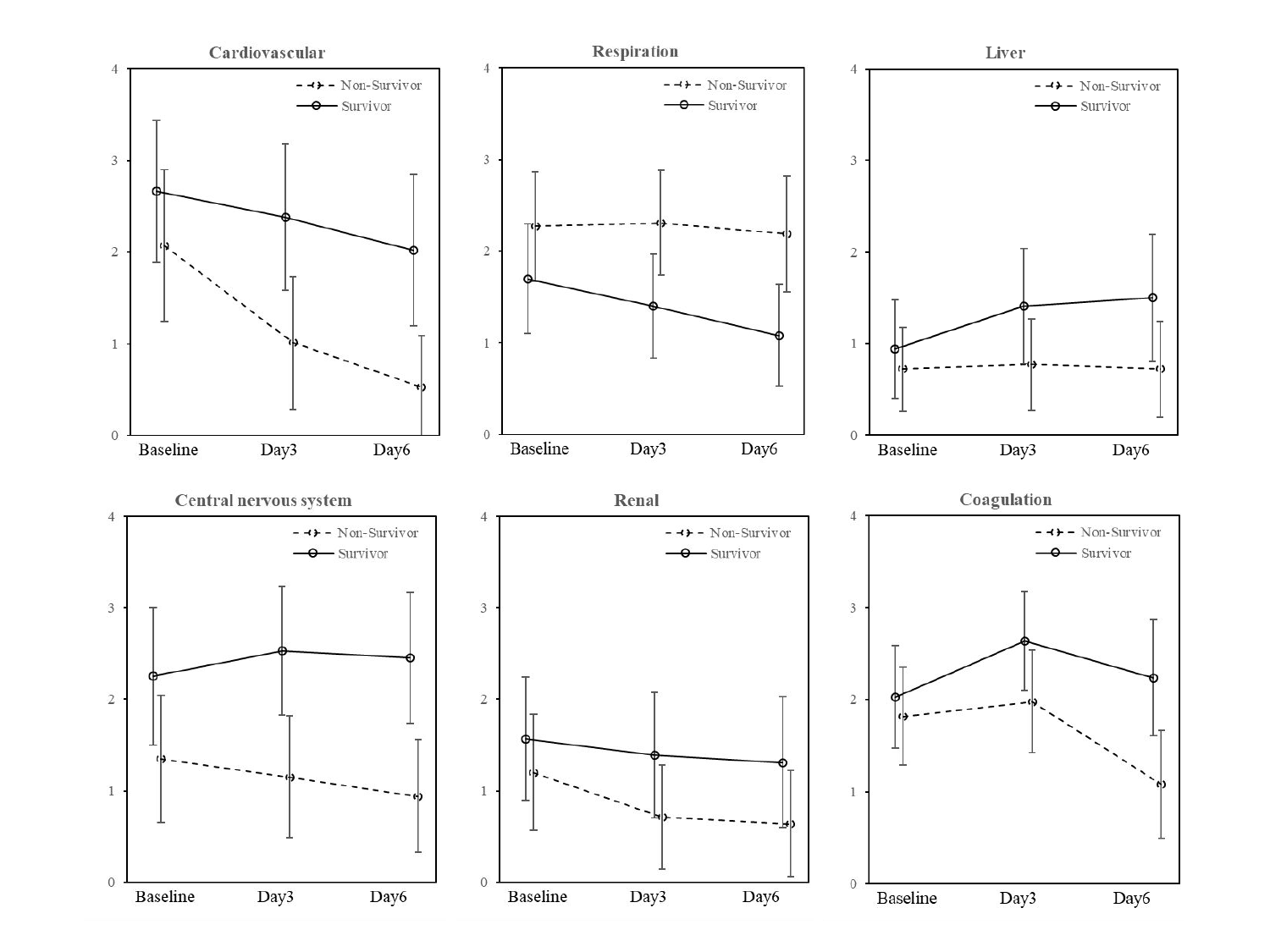

Supplement: Supplementary file 3 — Supplementary Figure 3. [file 41598_2023_49855_MOESM3_ESM.pptx]
